# Association Between Serum Insulin-Like Growth Factor 1 Levels and the Clinical Symptoms of Chronic Schizophrenia: Preliminary Findings

**DOI:** 10.3389/fpsyt.2021.653802

**Published:** 2021-03-03

**Authors:** Naomichi Okamoto, Koji Yoshino, Shogo Kitagawa, Rintaro Fujii, Shinsuke Hamada, Atsuko Ikenouchi, Yuki Konishi, Nobuhisa Ueda, Yoshinori Eto, Yasunari Tsutsumi, Reiji Yoshimura

**Affiliations:** ^1^Department of Psychiatry, University of Occupational and Environmental Health, Kitakyushu, Japan; ^2^Komine Eto Hospital, Kitakyushu, Japan; ^3^Tsutsumi Hospital, Kitakyushu, Japan

**Keywords:** schizophrenia, insulin-like growth factor 1, growth hormone, diabetes mellitus, clinical symptoms

## Abstract

**Purpose:** Insulin-like growth factor 1 (IGF-1) is a trophic mediator that is regulated by growth hormone and associated with the proliferation, development, and growth of neural cells. IGF-1 may be associated with the pathophysiology of schizophrenia, but this association remains controversial. This study aimed to investigate the relationship between serum IGF-1 levels and psychiatric symptoms in patients with chronic schizophrenia.

**Patients and Methods:** A total of 65 patients were recruited from the University of Occupational and Environmental Health, Komine Eto Hospital, Moji Matsugae Hospital, Shin-Moji Hospital, and Tsutsumi Hospital in Kitakyushu between September 2019 and June 2020. Further, 20 healthy age- and sex-matched control participants were recruited from the Komine Eto Hospital and the University of Occupational and Environmental Health. Patients with schizophrenia were assessed using the Positive and Negative Syndrome Scale (PANSS) and the Drug-Induced Extrapyramidal Symptoms Scale. Serum levels of free plus albumin-bound IGF-1 (IGF-1) were measured by immunoradiometric assay. The measurements were performed using antibody beads for bound/free separation. Associations between serum IGF-1 levels and the PANSS scores were determined. We also examined the associations between serum IGF-1 levels and diabetes, antipsychotic drug use, and disease duration.

**Results:** No significant difference was found in the serum IGF-1 level between patients with schizophrenia and healthy controls. Serum IGF-1 levels were significantly negatively correlated with the PANSS total score (*R*^2^ = 0.06, *p* = 0.015) and PANSS general score (*R*^2^ = 0.088, *p* = 0.008), but not with the PANSS positive scores and PANSS negative scores. Serum IGF-1 levels were not related to the prevalence of diabetes (*p* = 0.64). However, a significant correlation was observed between serum IGF-1 levels and age (*B* = −1.88, *p* < 0.0001). Serum IGF-1 levels could not distinguish patients with schizophrenia and healthy controls.

**Conclusion:** The association between serum IGF-1 levels and psychiatric symptoms may be complicated in patients with chronic schizophrenia.

## Introduction

Schizophrenia is a heterogeneous disorder with positive symptoms (delusions, hallucinations, thought disorders), negative symptoms (anhedonia, avolition, social withdrawal, and poverty of thought), and cognitive dysfunction, the origins of which appear to lie in the genetic and/or environmental disruption of brain development. The dopamine hypothesis, which states that dysregulation of the dopaminergic system is etiologic for schizophrenia, is one of the most enduring biological theories in psychiatry (1). There is strong evidence that implicates dysfunction of dopaminergic neurotransmission in the genesis of psychotic symptoms; abnormal glutamate signaling is implicated as well and may contribute to the negative and cognitive symptoms of schizophrenia. However, the current understanding of the neurobiology of schizophrenia remains largely incomplete (2, 3).

Insulin-like growth factor 1 (IGF-1) is a trophic mediator regulated by growth hormone (GH), which may be free or bound to binding proteins. IGF-1 is the most abundant binding protein in human blood and prolongs the half-life of IGF-1. IGF-1 regulates the proliferation, development, and growth of neural cells (4, 5) and plays a particularly important role in the development of psychiatric symptoms through neuronal growth and development, including neurogenesis and synaptic development (6). This peptide is also involved in the pathogenesis and evolution of psychiatric disorders, including major depression and schizophrenia, in preclinical (7, 8) and clinical studies (9–11). There are also indications that signaling pathways, including the IGF-1 pathway, may be involved in schizophrenia and other disorders treated with antipsychotic drugs (12). However, the precise relationship between IGF-1 and schizophrenia remains unknown.

Therefore, the aim of this study was to investigate the relationship between serum levels of free plus albumin-bound IGF-1 and psychiatric symptoms in patients with chronic schizophrenia. We also aimed to examine the associations between serum IGF-1 levels and diabetes, antipsychotic drugs, and disease duration.

## Methods

### Ethics Statement

The study protocol was approved by the Ethics Committee of the University of Occupational and Environmental Health, Kitakyushu, Japan (UOEHCRB19-024). All participants signed an informed consent document, in which the protocol and potential risks of the study were explained.

### Participants

Patients were recruited from the University of Occupational and Environmental Health, Komine Eto Hospital, Moji Matsugae Hospital, Shin-Moji Hospital, and Tsutsumi Hospital in Kitakyushu between September 2019 and June 2020. Healthy control participants were also recruited from the Komine Eto Hospital and the University of Occupational and Environmental Health. All patients fulfilled the diagnostic criteria for schizophrenia outlined in the structured clinical interview of the Diagnostic and Statistical Manual for Mental Disorders, Fifth Edition. Patients with a history of neurological disorders, substance use, and/or other psychiatric disorders were excluded. Healthy participants were identified as those who did not fulfill any of the diagnostic criteria in the Diagnostic and Statistical Manual for Mental Disorders, Fifth Edition (13). Chronic schizophrenia was defined as a duration of illness of ≥10 years. The controls were age- and sex-matched the patients as much as possible and had no pre-specified medical conditions. The relationship between IGF-1 and schizophrenia symptoms was evaluated. We also examined the associations between serum IGF-1 levels and diabetes, antipsychotic drug use, and duration of illness.

### Clinical Evaluations

Patients with schizophrenia were assessed using the Positive and Negative Syndrome Scale (PANSS) (14), Drug-Induced Extrapyramidal Symptoms Scale (15), and blood sampling.

#### PANSS

##### Drug-Induced Extrapyramidal Symptoms Scale

*Blood sampling.* Blood sampling was performed between 7 a.m. and 9 a.m. The participants fasted and rested for at least 30 min before blood samples were drawn. Samples were sent from the University of Occupational and Environmental Health to SRL Inc. (SRL, Kitakyushu, Japan). At SRL, IGF-1 levels in the samples were measured by immunoradiometric assay. The measurements were performed using antibody beads for bound/free separation. IGF-1 antibody beads were reacted with diluted samples of standard IGF-1 reagent, control reagent, or test serum. Thereafter, an iodized IGF-1 antibody (125I) reagent was added to form a complex of bead-solidified IGF-1-IGF-1-iodized IGF-1 antibody (125I), and the radioactivity of the bound was measured after removal of the internal solution. The concentrations of IGF-1 in the control reagent and test sera were determined from standard curves obtained from the standard IGF-1 reagent.

### Data Analysis

All statistical analyses were performed using EZR software version 1.50, which was used to calculate R (16).

Patients with schizophrenia were compared to healthy control participants. Male patients with schizophrenia were also compared to female patients with schizophrenia. We used the Student's *t*-test to compare continuous variables after confirming the equality of variance for variables that followed a normal distribution, and the Mann-Whitney *U*-test was used for variables that did not follow a normal distribution. Fisher's exact test was used to compare nominal variables.

Multivariate regression analysis was performed in the schizophrenia group to determine the relationships between serum IGF-1 levels and clinical symptoms of schizophrenia (total, positive, negative, and general scores), CP total, and disease duration. Because the IGF-1 level is affected by age, sex, and height/weight, we adjusted for age, sex, and body mass index as confounding factors. In addition, logistic regression analysis was conducted in the schizophrenia group, with the prevalence of diabetes as the objective variable. Data values are expressed as the mean (standard deviation) or median (interquartile range). All statistical analyses were two-tailed, and significance was defined as a *p*-value of <0.05.

## Results

### Background Characteristics

A total of 65 patients with schizophrenia and 20 healthy participants were included in the study. The background and clinical characteristics of patients with schizophrenia and healthy controls are presented in Table 1.

**Table 1:** Clinical and demographic characteristics.

	**Patients with schizophrenia (*n* = 65)**	**Healthy controls (*n* = 20)**	***p*-value**
Age (years), mean (SD)	49 (10)	46 (7.4)	0.35
Male sex, n (%)	33 (51%)	8 (40%)	0.45
BMI (kg/m^2^), mean (SD)	22 (4.9)	23 (3.8)	0.89
DM, n (%)	5 (7.7%)	1 (5.0%)	1.00
IGF-1 (ng/mL), mean (SD)	109 (38)	120 (39)	0.27
PANSS total, mean (SD)	87 (20)	–	–
PANSS positive, mean (SD)	20 (5.3)	–	–
PANSS negative, mean (SD)	23 (6.8)	–	–
PANSS general, median [IQR]	43 [38–49]	–	–
DIEPSS, median [IQR]	5 (3–9)	–	–
CP total, mean (SD)	732 (390)	–	–

*SD, standard deviation; IQR, inter-quartile range; DM, diabetes mellitus; BMI, body mass index; IGF-1, insulin-like growth factor 1; PANSS, Positive and Negative Syndrome Scale; DIEPSS, Drug-Induced Extrapyramidal Symptoms Scale; CP, chlorpromazine*.

**Figure 1:**
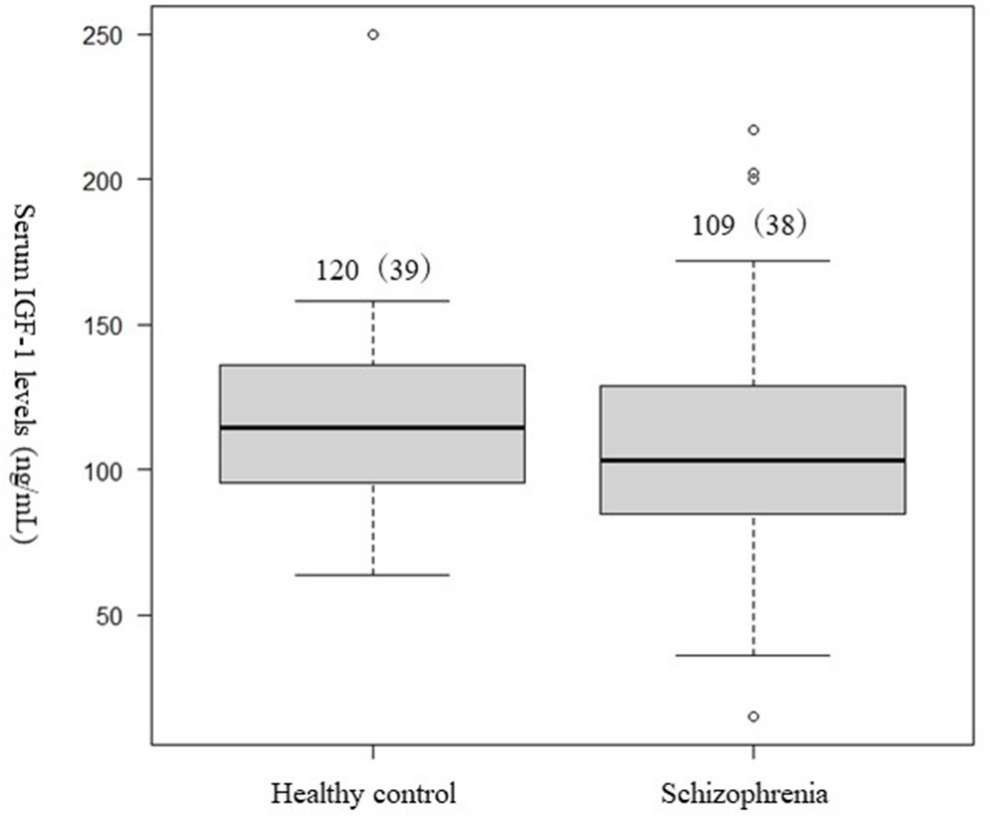
Differences in the serum (IGF-1) levels between patients with schizophrenia and healthy controls. Student's *t*-test was used to compared the schizophrenia group and healthy controls. Not significant difference was observed in serum IGF-1 levels between patients with schizophrenia and the healthy controls (*p* = 0.27).

No significant difference was observed in the serum IGF-1 levels between patients with schizophrenia and healthy controls (Table 1, Figure 1).

### Serum IGF-1 or CP Total and Disease Duration

There were no significant associations between serum IGF-1 and CP total (*R*^2^ = 0.22, *p* = 0.81) or disease duration (*R*^2^ = 0.23, *p* = 0.63).

### Serum IGF-1 and the PANSS Scores

**Figure 2:**
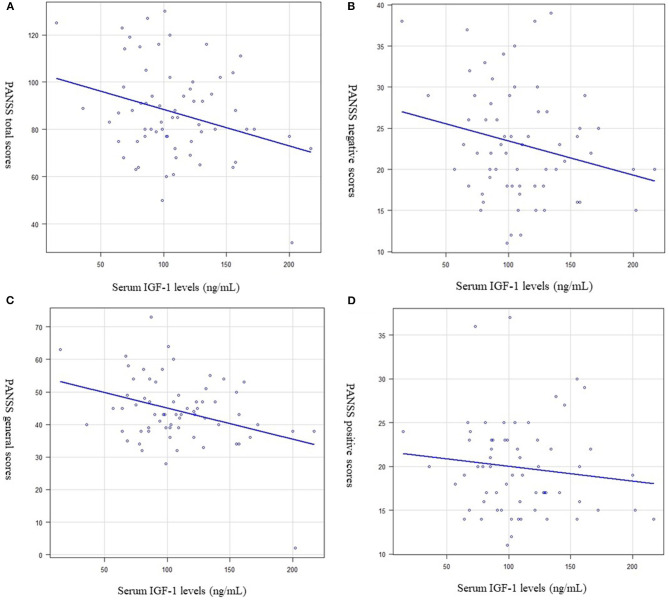
Relationship between the serum (IGF-1) levels and psychiatric symptoms (Multiple regression analysis was used, at adjusted for age, sex, and BMI as independent factors). **(A)** Serum IGF-1 levels and the Positive and Negative Syndrome Scale (PANSS) total score. Serum IGF-1 levels were significantly negatively correlated with PANSS total scores. (*R*^2^ = 0.06, *p* = 0.015). **(B)** Serum IGF-1 levels and the PANSS general score. Serum IGF-1 levels were significantly negatively correlated with PANSS general scores (*R*^2^ = 0.088, *p* = 0.008). **(C)** Serum IGF-1 levels and the PANSS positive score. Serum IGF-1 levels were not significantly correlated with PANSS positive scores. (*R*^2^ = −0.014, *p* = 0.16). **(D)** Serum IGF-1 levels and the PANSS negative score. Serum IGF-1 levels were not significantly correlated with PANSS negative scores. (*R*^2^ = 0.022, *p* = 0.06).

Serum IGF-1 levels were significantly negatively correlated with the PANSS total (PANSS-T) score (*R*^2^ = 0.06, *p* = 0.015, Figure 2A) and PANSS general (PANSS-G) score (*R*^2^ = 0.088, *p* = 0.008, Figure 2B). However, serum IGF-1 levels were not significantly correlated with the PANSS positive (*R*^2^ = −0.014, *p* = 0.16, Figure 2C) or negative (*R*^2^ = 0.022, *p* = 0.06) scores (Figure 2D).

**Figure 3:**
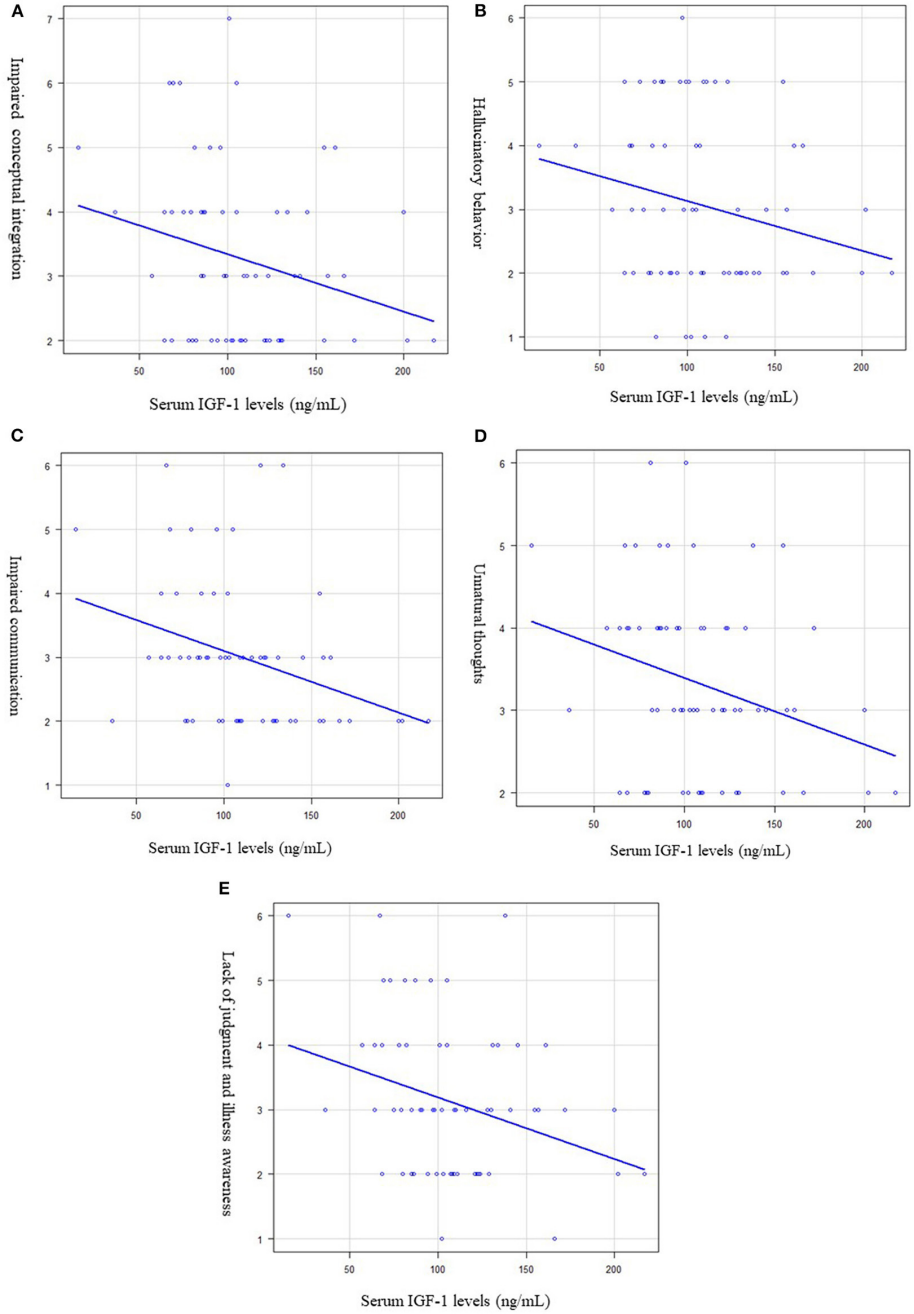
Relationship between the serum (IGF-1) levels and each PANSS item. Multiple regression analysis was used, at adjusted for age, sex, and BMI as independent factors. **(A)** Serum IGF-1 levels and impaired conceptual integration. Serum IGF-1 levels were significantly negatively correlated with impaired conceptual integration. (*R*^2^ = 0.053, *p* = 0.011). **(B)** Serum IGF-1 levels and hallucinatory behavior. Serum IGF-1 levels were significantly negatively correlated with hallucinatory behavior. (*R*^2^ = 0.008, *p* = 0.038). **(C)** Serum IGF-1 levels and impaired communication. Serum IGF-1 levels were significantly negatively correlated with impaired communication. (*R*^2^ = 0.053, *p* = 0.021). **(D)** Serum IGF-1 levels and unnatural thoughts. Serum IGF-1 levels were significantly negatively correlated with unnatural thoughts. (*R*^2^ = 0.027, *p* = 0.034). **(E)** Serum IGF-1 levels and lack of judgement and illness awareness. Serum IGF-1 levels were significantly negatively correlated with lack of judgement and illness awareness. (*R*^2^ = 0.048, *p* = 0.044).

We further analyzed the correlation between serum IGF-1 levels and each PANSS item. Significant negative correlations were observed between serum levels of IGF-1 and impaired conceptual integration (*R*^2^ = 0.053, *p* = 0.011, Figure 3A), hallucinatory behavior (*R*^2^ = 0.008, *p* = 0.038, Figure 3B), impaired communication (*R*^2^ = 0.053, *p* = 0.021, Figure 3C), unnatural thoughts (*R*^2^ = 0.027, *p* = 0.034, Figure 3D), and lack of judgment and illness awareness (*R*^2^ = 0.048, *p* = 0.044, Figure 3E).

### Serum IGF-1, Disease Prevalence, and Age

Logistic regression analysis revealed no significant associations between IGF-1 levels and the prevalence of diabetes (*p* = 0.64) (Table 2) or between serum IGF-1 levels and a diagnosis of schizophrenia (Table 3). However, multiple regression analysis indicated a significant correlation between serum IGF-1 levels and age (*B* = −1.88, *p* < 0.0001) (Table 4).

**Table 2:** Logistic regression analysis with prevalence of diabetes and objective variables.

	**OR (95% CI)**	***p*-value**
Age	0.98 (0.78–1.23)	0.87
Male sex	3.03 (0.29–31.8)	0.36
BMI	1.02 (0.88–1.29)	0.84
IGF-1	0.99 (0.96–1.02)	0.64
Disease period	1.06 (0.88–1.29)	0.53
CP total	0.99 (0.99–1.00)	0.44

*OR, odds ratio; CI, confidence interval; BMI, body mass index; IGF-1, insulin-like growth factor 1; CP, chlorpromazine*.

**Table 3:** Logistic regression analysis with phenotype (schizophrenia or control) and objective variables.

	**OR (95% CI)**	***P*-value**
Age	1.02 (0.96~1.09)	0.54
Male sex	1.12 (0.38~3.33)	0.84
BMI	0.96 (0.85~1.09)	0.56
IGF-1	0.99 (0.98~1.01)	0.45

*OR, odds ratio; CI, confidence interval; BMI, body mass index; IGF-1, insulin-like growth factor 1*.

**Table 4:** Multiple regression analysis with IGF-1 levels and objective variables.

	**B (95% CI)**	***p*-value**
Age	−1.88 (−2.69~1.07)	<0.001
Male sex	1.59 (−13.9~17.1)	0.84
BMI	0.63 (−1.05~2.30)	0.46
Schizophrenia group	−7.04 (−25.6~11.5)	0.45

*B, partial regression coefficient; CI, confidence interval; BMI, body mass index; IGF-1, insulin-like growth factor 1*.

## Discussion

In this study, we investigated the relationships between serum IGF-1 levels and psychiatric symptoms in patients with chronic schizophrenia and analyzed the association between serum IGF-1 levels and diabetes, antipsychotic drug use, and duration of illness. The results showed that serum IGF-1 levels were related to the PANSS scores in patients with chronic schizophrenia; we observed a significant negative correlation between serum levels and PANSS-T and PANSS-G scores. Moreover, significant negative correlations were observed between serum IGF-1 levels and the PANSS subscales, including impaired conceptual integration, hallucinatory behavior, dysphoria, unnatural thinking, and impaired judgment and awareness. These results indicate that serum IGF-1 levels are partially related to the broad symptoms of schizophrenia, including positive, negative, and general symptoms. Previous studies have reported that IGF-1 levels are reduced in schizophrenia (17), and that the reduced IGF-1 levels may be involved in the pathogenesis, particularly in the negative symptoms, of schizophrenia (18). However, another study revealed elevated IGF-1 levels in patients with schizophrenia (10, 17), but this result is not in accordance with the literature. Petrikis et al. (11) reported elevated levels of IGF-1 in drug-naive patients with psychosis, including schizophrenia. Therefore, the use of medications may have affected the study results, including ours. Considering the variations in these findings, the blood IGF-1 levels in patients with schizophrenia are still controversial. The reason for this discrepancy remains unknown. However, the heterogeneity of the patients with schizophrenia enrolled in the present study and previous studies is a plausible explanation.

It is also reported that reduced IGF-1 is a cause of diabetes mellitus (DM) *via* the GH/IGF-1 axis (17). GH stimulates the production of IGF-1 in most tissues, and together, GH and IGF-1 exert powerful collective actions on fat, protein, and glucose metabolism. The results of the present study indicate that serum IGF-1 levels function independently in patients with chronic schizophrenia. The reason for the alteration in IGF-1 levels in patients with schizophrenia is not clear; however, it was not changed in the present study. GH, glucocorticoids, and nutrition may influence the IGF-1 levels (19). Some studies have reported an association between IGF-1 polymorphisms and serum IGF-1 levels in patients with schizophrenia (20). IGF-1 influences all major neuronal cell types, including neural stem cells, neurons, and glial cells in the brain (4). IGF-1 also plays an important role in neuronal synaptic activity in the proliferation of GABAergic agents, glutamatergic neurons (21), and neurogenesis in the hippocampus (22). Therefore, it is plausible that synaptogenesis induced by IGF-1 underlies the association between serum IGF-1 levels and the PANSS scores of patients with schizophrenia. The relationship between serum IGF-1 levels and psychopathology in patients with schizophrenia thus appears to be complicated.

In contrast, studies on glucose intolerance and IGF-1 have been conducted using knockout mice lacking the *insulin receptor substrate 2 (IRS2)* gene (23). IRS2 is an IGF-1 receptor and is widely expressed throughout the body and brain (22). Systemic IRS2-deficient mice exhibit a striking phenotype of insulin resistance, severe diabetes, and juvenile lethality (24). Thus, we hypothesized that serum IGF-1 levels are affected by the DM status in patients with schizophrenia. However, multivariate logistic regression analysis did not show a clear and significant relationship between serum IGF-1 levels and the prevalence of diabetes.

This study has some limitations. First, the sample size was small, and all patients with schizophrenia were in the chronic stage and had been receiving several antipsychotic drugs, which could have affected the serum IGF-1 levels. Second, metformin (25), fasting (26), and physical exercise (27) are known to influence serum IGF-1 levels. Third, we performed multiple statistical tests. We are currently planning a study with a larger sample of first-episode, drug-naive patients with schizophrenia, controlling these confounding factors, which will yield more accurate results of the relationship between serum IGF-1 levels, and clinical symptomatology in schizophrenia without drug-related influences.

In conclusion, our study indicated that serum IGF-1 levels were related to each item of the PANSS subscales. Notably, the serum IGF-1 levels did not differ between the schizophrenia and healthy control groups. In addition, we found that serum IGF-1 levels were not related to the prevalence of DM.

## Data Availability Statement

The raw data supporting the conclusions of this article will be made available by the authors, without undue reservation.

## Ethics Statement

The studies involving human participants were reviewed and approved by the study protocol was approved by the Ethics Committee of the University of Occupational and Environmental Health, Kitakyushu, Japan. All the participants who were enrolled in the study signed an informed consent document, in which the protocol and potential risks of the study were explained. The patients/participants provided their written informed consent to participate in this study.

## Author Contributions

NO and RY: conceptualization. NO: methodology, software, and visualization. NO, AI, YK, and RY: validation and writing—original draft preparation. NO, KY, SK, RF, SH, AI, YK, NU, YE, and YT: data curation. AI, YK, and RY: writing—review and editing and supervision. RY: funding acquisition. All authors: have read and agreed to the published version of the manuscript.

## Conflict of Interest

The authors declare that the research was conducted in the absence of any commercial or financial relationships that could be construed as a potential conflict of interest.
